# Culturally appropriate methodology in obtaining a representative sample of South Australian Aboriginal adults for a cross-sectional population health study: challenges and resolutions

**DOI:** 10.1186/s13104-015-1080-5

**Published:** 2015-05-19

**Authors:** Tania Marin, Anne Winifred Taylor, Eleonora Dal Grande, Jodie Avery, Graeme Tucker, Kim Morey

**Affiliations:** The University of Adelaide, Adelaide, South Australia Australia; South Australian Health and Medical Research Institute (SAHMRI), Adelaide, South Australia Australia

**Keywords:** Aboriginal health, Methodology, Recruitment, Population survey, Cultural appropriateness

## Abstract

**Background:**

The considerably lower average life expectancy of Aboriginal and Torres Strait Islander Australians, compared with non-Aboriginal and non-Torres Strait Islander Australians, has been widely reported. Prevalence data for chronic disease and health risk factors are needed to provide evidence based estimates for Australian Aboriginal and Torres Strait Islanders population health planning. Representative surveys for these populations are difficult due to complex methodology. The focus of this paper is to describe in detail the methodological challenges and resolutions of a representative South Australian Aboriginal population-based health survey.

**Methods:**

Using a stratified multi-stage sampling methodology based on the Australian Bureau of Statistics 2006 Census with culturally appropriate and epidemiological rigorous methods, 11,428 randomly selected dwellings were approached from a total of 209 census collection districts. All persons eligible for the survey identified as Aboriginal and/or Torres Strait Islander and were selected from dwellings identified as having one or more Aboriginal person(s) living there at the time of the survey.

**Results:**

Overall, the 399 interviews from an eligible sample of 691 SA Aboriginal adults yielded a response rate of 57.7%. These face-to-face interviews were conducted by ten interviewers retained from a total of 27 trained Aboriginal interviewers. Challenges were found in three main areas: identification and recruitment of participants; interviewer recruitment and retainment; and using appropriate engagement with communities. These challenges were resolved, or at least mainly overcome, by following local protocols with communities and their representatives, and reaching agreement on the process of research for Aboriginal people.

**Conclusions:**

Obtaining a representative sample of Aboriginal participants in a culturally appropriate way was methodologically challenging and required high levels of commitment and resources. Adhering to these principles has resulted in a rich and unique data set that provides an overview of the self-reported health status for Aboriginal people living in South Australia. This process provides some important principles to be followed when engaging with Aboriginal people and their communities for the purpose of health research.

## Background

The state of Australian Aboriginal and Torres Strait Islander health has been extensively documented [1-8]. Considerably lower average life expectancy for Aboriginal and Torres Strait Islander Australians, compared with non-Aboriginal and non-Torres Strait Islander Australians, has been widely reported [4,6,9,10]. Much of the health literature focuses on chronic illness such as: diabetes [11-13], kidney disease [11,14-16], high blood pressure [11,16], and cardiovascular disease [12]; and the effects of living in urban versus remote and very remote communities [17,18]. It is well recognised that significant improvements in Aboriginal and Torres Strait Islander health outcomes are still to be made [19].

The literature provides examples of Aboriginal and Torres Strait Islander health research studies in Australia ranging from large national population data collections [1,20] to those that employ methodologies such as convenience sampling or snowballing to achieve their aims [21-24]. Reliable representative population health data for these populations, essential for program and policy planning at state or regional level, are not available [25]. Although data are collected regularly on a national level, these collections do not provide a timely and clear picture of Aboriginal and Torres Strait Islander health at a state level [26].

In the past decade, a number of participatory health research studies for Aboriginal populations have emerged both globally [27] and in Australia [28]. One example is the study of urban young people by an Aboriginal Community Controlled Health Organisation in the state of Victoria [29]. This study is useful in terms of contributing to the knowledge of research approaches that addressed cultural issues identified in Aboriginal and Torres Strait Islander communities. A significant outcome of this study was the establishment of a cohort for a longitudinal study [30]; however, the scope was limited and not representative of a large population in order to allow for comparison to other Aboriginal populations. In 2000, the Western Australian Aboriginal Child Health Survey (WAACHS) [31,32], a cross-sectional study of approximately 5,300 interviews, collected data for randomly selected Aboriginal children under the age of 18 years. First proposed in 1991 during the development of the Western Australian Child Health Survey [33], the WAACHS was undertaken between May 2000 and June 2002, following a long consultation period with Aboriginal and Torres Strait Islander leaders and communities. An undertaking of this size was the first of its kind outside of federally funded data collections: the methodology for this survey has been described in detail elsewhere [34].

Neither of the aforementioned studies collected data for Australian Aboriginal adult populations, and representative data for these populations could not be found in the literature. Access to information identifying Aboriginal and/or Torres Strait Islander dwellings is not publically available, making random selection for large population household surveys extremely difficult [35]. A high level of screening is needed to find dwellings where Aboriginal and/or Torres Strait Islanders reside using available dwelling statistics, especially in city environments [36,37]. A further gap in the literature was identified regarding the need to engage with South Australian Aboriginal communities and their representatives; knowing when and how to seek advice on local protocols and a need for agreement on the right way to undertake culturally appropriate research in Aboriginal populations.

In 2009, the South Australian Department for Health and Ageing (SA Health), contracted Population Research and Outcome Studies, The University of Adelaide, South Australia (SA), to develop and manage a representative South Australian Aboriginal population health survey. The term ‘Aboriginal’ is used respectfully for the remainder of this document as an all-encompassing term for Aboriginal and/or Torres Strait Islander Peoples reflecting the early community consultation that suggested a preference for ‘Aboriginal’ to be used.

The aim of the South Australian Aboriginal Health Survey (SAAHS) was to collect data for the SA Aboriginal adult population using a culturally appropriate methodology to produce prevalence estimates for chronic illness and risk factors. The project was funded by State government under the Council of Australian Governments National Partnership Agreement on Closing the Gap for Indigenous Health Outcomes [5]. Questions focussed on social determinants of health, chronic disease, risk factors, and health service access: all current major Closing the Gap policy drivers. Planning and development occurred locally over an eight month period of collaboration and consultation, under the guidance of an Advisory Committee (AC) consisting of both Aboriginal and non-Aboriginal representatives. Representatives came from government (SA Health, the Australian Bureau of Statistics (ABS), and the Office of Aboriginal and Torres Strait Islander Health) and non-government (Aboriginal Health Council of SA and Cancer Council SA) organisations. This consultative process originally included the Anangu Pitjantjatjara Yankunytjatjara (APY) Lands (Aboriginal owned Lands in the north of SA); however, as a result of advice from local health personnel and out of respect for cultural and organisational protocols, it became inappropriate for the research team to survey APY Land’s residents and data collection in this region did not go ahead.

The SAAHS AC steered the development of the methodology to adequately and properly apply sampling techniques to enable extrapolation of the results to the larger SA Aboriginal population. The aim of this paper is to describe in detail the methodological challenges and associated resolutions found with this survey methodology.

## Methods

The SAAHS methodology was designed around evidence-based data collection methods for population health surveys and culturally appropriate protocols for engaging Aboriginal people. This design used rigorously sound epidemiological methods whilst ensuring as little impact to SA Aboriginal communities and community members as possible.

### Consultation

An eight month consultation was undertaken between SAAHS AC members, Aboriginal community members and Elders, and primary SA stakeholders in Aboriginal health. This process was informed by a part Aboriginal governance structure, advice from the government and community controlled health sector, and Aboriginal leaders. The consultation involved discussions on community priorities for data collection, current major policy drivers for the SA Aboriginal adult population, and logistics of engaging Aboriginal interviewers and participants. Further discussions focussed on cultural appropriateness and sensitivity issues for the wording, structure and question sequence of the questionnaire.

The SAAHS was committed to adhering to evidence based population research methods with the ability to adapt strategies to include cultural respectfulness [38], including; meeting with community Elders before conducting research, employing local Aboriginal people to conduct the interviews, and providing support to conduct interviews according to local needs and protocols.

### Recruiting interviewers

All interviewers were Aboriginal and fully trained to carry out the SAAHS data collection. Training was undertaken by SAAHS coordinators and consisted of a step-by-step explanation of the questionnaire, rights and responsibilities of interviewers and participants, and administrative procedures associated with the project (i.e. timesheets, reimbursement of out of pocket expenses, etc.). It was reiterated throughout the project that interviewer safety was at all times paramount: the interviewer’s discretion on these matters was not questioned.

There were two structured training days held in metropolitan Adelaide (one north and one central), one in the remote west of SA, and one in Port Augusta in the north of SA. Additionally, interviewers taken on as the project progressed were trained one-on-one. A total of 27 Aboriginal interviewers were recruited and fully trained. Interviewers were of different ages and gender, and lived in a range of areas (metropolitan, rural, and remote); they represented different family and language groups with varied networks.

### Data collection

Data collection began in November 2010 and concluded in October 2011. Data were collected using personal face-to-face interviewing techniques by Aboriginal interviewers who worked in pairs where possible; one male and one female. Participants were offered a choice of gender of interviewer to ensure cultural and sensitivity protocols were respected [38]. The interviews were conducted in the respondent’s home, unless a more suitable venue was requested. In remote communities, interviewers were often accompanied by community facilitators, who assisted in the conduct and completion of the interviews, explained the purpose of the survey to respondents, introduced the interviewers, assisted in identifying the residents of a household and in locating residents who were not at home, and assisted the respondent’s understanding of the questions.

### Sample size

Epi-Info [39] was used to calculate the sample size required. The sample size calculation was based on 15% of Aboriginal adults (aged 15 years and over) with diabetes [40], an Aboriginal population of 16,265, +/− 3.0% error, and a 95% confidence interval. Based on these requirements, a minimum sample size of 527 Aboriginal people was determined.

### Stage 1: sampling frame

The sampling frame was compiled using SA Collection Districts (CDs) (n = 3,247) from the ABS 2006 Census numbers of Aboriginal adult usual residents (URs) (n = 16,525). In an attempt to increase the chances of finding Aboriginal people, all CDs with a count of less than ten Aboriginal URs and a ratio of Aboriginal people to total number of dwellings less than 0.05 but greater than zero, were discarded. These limitations provided a sampling frame of 73.5% of the total SA Aboriginal population living in private dwellings: 60.4% of metropolitan; 74.1% of rural; and 91.0% of remote; living in 875 CDs.

### Stage 2: probability sampling of CDs

The sample was stratified by remoteness using the classifications of the five ABS remoteness area classifications [41] grouped into three regions, with every CD classified into one of those regions, using the overall state distribution of 47% in metropolitan Adelaide, 32% in rural areas, and the remaining 21% in remote areas and discrete communities. The Statistical Package for the Social Sciences (SPSS) [42] was used to create a random sample of CDs from those eligible for selection, based on the sample size calculation of 1,034 (allowing for a 50% refusal rate). A CD with a higher ratio of Aboriginal people to total number of dwellings had a higher probability of being selected, and some CDs were selected more than once as a consequence. This resulted in a sample of 193 unique CDs from 232 selections.

### Stage 3: selection of eligible dwellings

Maps of each selected CD were obtained from the ABS and a structured contact screening procedure designed to identify eligible dwellings. A path was created on each map by keeping to the left of a street, turning left at corners where possible, and continuing in this way as far as possible to pass every house in the CD. When a ‘dead end’ was encountered, the process started again at another randomly chosen start point. Starting from a previously assigned randomly generated start point between one and three, each third house was selected providing a list of randomly chosen dwellings in each CD. It was not known which dwellings were home to eligible people (Aboriginal and aged 15 years or over), and therefore, each selected dwelling was approached to ascertain eligibility. Only private dwellings (not institutions such as hospitals, private nursing homes, or prisons), were included.

Fully trained Aboriginal and non-Aboriginal health research workers were given contact sheets, with dwellings ordered numerically along the path, to keep record of contact attempts. To decrease clustering in metropolitan and rural CDs, the aim was to identify up to four eligible persons per CD, and ten eligible persons in each remote CD and door-knocking ceased when this number of eligible adults had been identified from at least two dwellings along the path for each CD. This meant the total number of dwellings contacted varied for each CD. Hence, all dwellings that were approached (along the path) were included in the sampling frame, while the remaining dwellings were not. If a dwelling was approached at least once but there was no answer (eligibility unknown) but four eligible adults had been identified from at least two dwellings along the path, the dwelling was continued to be approached (up to five times) to establish eligibility. For example, dwellings #1 to #10 were approached in one day, and dwellings #4 and #10 were identified having at least four eligible adults. However, dwellings #2, #6 and #7 resulted in no one answering. This means that these three dwellings were part of the selection and as such were approached at least five times until their eligibility was determined. If any of these dwellings had at least one Aboriginal adult resident then they were included in the study. To ascertain Aboriginality, the standard question from the National Best Practice guidelines for collecting Indigenous status in health data sets was used [43], adapted slightly to include the age criteria.

Where an Aboriginal household was identified by an Aboriginal researcher, any eligible residents were offered the opportunity to undertake the interview then and there, or book a time to be followed up. Where a non-Aboriginal researcher identified an eligible residence, they asked the person at the door whether they would be willing to speak to an Aboriginal person regarding an Aboriginal health survey.

### Stage 4: selection of eligible persons

Where a dwelling contained more than one Aboriginal adult (aged 15 years or over), all people identifying as Aboriginal were selected to participate in the survey; this included usual and temporary residents. Usual residents were defined as having lived in the dwelling for six months or more; temporary residents were defined as those who had been living in the dwelling for more than one month but less than six months. There was no replacement made for refusals or non-contactable persons. When the required number of eligible persons was reached (regardless of whether people participated in the survey) that CD was deemed to be completed.

### Data analyses

All data were inputted into Microsoft Excel and a 10% quality check performed. Data were then imported into SPSS version 18.0 and weighted by age, sex and remoteness area to the ABS 2006 estimated resident population (excluding APY Lands). Two weighting factors were made: the first providing the best estimates for overall SA (used for these analyses); and the second providing the best estimates for each of the three SA regions; metropolitan Adelaide, rural SA, and remote SA. Due to the inability to collect data from the APY Lands, all estimates provided here are for SA excluding this region. Statistical analyses were carried out using SPSS version 18.0 and χ^2^ testing used to compare differences in categorical variables. Statistical significance was set at p ≤ 0.05 (two sided).

### Ethics

The SAAHS was approved by both the Aboriginal Health Research Ethics Committee and the SA Health Human Research Ethics Committee.

## Results

Overall, the 399 interviews, from an eligible sample of 691 SA Aboriginal adults, yielded a response rate of 57.7%. Sample losses were due to refusal (19.4%), respondents being repeatedly unavailable to interview (19.5%), illness, incapability to undertake interview, or having moved since first contact (3.3%). Interviews were conducted by ten interviewers retained from a total of 27 trained interviewers.

### Procedural

Challenges were found in three main areas: identification and recruitment of participants; interviewer recruitment and retainment; and appropriate engagement with communities.

The recruitment of participants was hindered by the complex methodology required to firstly identify eligible households. Over 11,000 dwellings were screened for eligibility. From this, a total of 345 dwellings were identified as having one or more Aboriginal adult (15 years of age or over) living there at the time of the approach. To manage the laborious task of identifying eligible households, and the disappointment being experienced by the initial Aboriginal interviewers employed who were not used to this work, a third party organisation was contracted to door-knock. Although a number of the original interviewers (eight out of ten) had left by this stage, the process was completed in time to meet project timelines.

Once contacted, eligible persons were often keen to participate and in most cases the other Aboriginal members of the household would also be interviewed. This was often the initiative of the interviewer at the time of the first interview.

From the ten interviewers initially trained and recruited only two went on to conduct interviews. The list of applicants was revisited and a second group of Aboriginal people from metropolitan, rural and remote areas trained as interviewers. This involved travel to rural and remote areas to train people face-to-face. In part, interviewer attrition was overcome by using constant communication between the SAAHS coordinators and the interviewers, and between the interviewers. Some interviewers were highly motivated; one interviewer conducted 100 interviews, while others needed more help and guidance. Interviewers were paired to overcome the issue of working alone in a household environment, and to give the participant a gender choice of interviewer (usually the pair consisted of one male and one female). By doing this the interviewers gave each other ongoing support and motivation. In one instance, the older interviewer took on a mentoring role for the younger.

Where it was possible for SAAHS coordinators to meet with interviewers locally, this proved to be a more successful way of engaging. Interviewers themselves often identified ways to achieve better results. For example, where previously collected telephone numbers were mobile (cell-phone) numbers, text messaging was used as a way for the interviewer to introduce themselves, addressing the common problem of people not answering phone-calls from unknown numbers.

### Cultural engagement

Appropriate engagement with community was initially a challenge for the project. Extensive consultation was used as the method to create relationships with Aboriginal community representatives and inform research practice. Agreements on the methods used for conducting research were reached and methodologies adapted to local protocols and acceptable conduct. The quality of the data was very much improved through this consultation process. The choice to use face-to-face interviewing and visiting people where they lived was seen as a respectful way to engage people in the health research process. When the first report was written [44], effort was made to make it publicly available. The decision to produce a series of easy to read pamphlets, highlighting the results from the survey for community, recognised the importance of reciprocity when undertaking health research in Aboriginal communities.

### Reliability

Table 1 shows the comparison between the unweighted sample from the SAAHS and the ABS 2006 Census for Population and Housing (ABS, 2006) for age, gender, and remoteness. To determine if the SAAHS respondents were different from the ABS Aboriginal Census population, we assessed if the Census population proportions lay outside of the estimated confidence interval from the SAAHS. There were no statistical differences found for age and gender, however, the SAAHS over-represents remote areas and under-estimates metropolitan Adelaide when compared to the ABS profile (Table 1).

**Table 1:** Comparison of demographic variables: ABS Census, ABS sampling frame, SAAHS, aged 15 years and over

	**ABS 2006 profile** ^**1**^		**ABS – SAAHS ineligible CDs** ^**2**^		**ABS – SAAHS eligible CDs** ^**2**^		**SAAHS** ^**3**^ **(2010–2011)**	
	**n**	**%**	**n**	**%**	**n**	**%**	**n**	**% (95% CI)**
**Sex**								
Male	7,079	48.4	2,154	50.2	5,555	46.7	189	47.4 (42.5 - 52.3)
Female	7,802	51.5	2,139	49.8	6,339	53.3	210	52.6 (47.7 - 57.5)
**Age group**								
15 – 24 yrs	4,606	31.0	1,265	29.5	3,746	31.5	109	27.3 (23.2 - 31.9)
25 – 34 yrs	3,185	21.4	886	20.6	2,640	22.2	89	22.3 (18.5 - 26.6)
35 – 44 yrs	3,012	20.2	814	19.0	2,458	20.7	88	22.1 (18.3 - 26.4)
45 – 54 yrs	2,172	14.6	677	15.8	1,610	13.5	54	13.5 (10.5 - 17.2)
> = 55 yrs	1,908	12.8	1,439	15.2	1,439	12.1	59	14.8 (10.5 - 17.2)
**Remoteness**								
Metropolitan	**7,777**	**52.3**	**3,080**	**71.8**	4,696	39.8	157	39.3 (34.7 - 44.2)
Rural	5,240	35.2	**1,278**	**25.1**	3,718	35.0	158	39.6 (34.9 - 44.5)
Remote	**1,866**	**12.5**	**132**	**3.1**	**3,040**	**25.5**	84	21.1 (17.3 - 25.3)
**Total**	**14,883**	**100.0**	**5,214**	**100.0**	**10,403**	**100.0**	**399**	**100.0**

Sources: ^1^ABS Census 2006 (South Australia excluding APY Lands); ^2^ABS Census 2006 (South Australia includes APY Lands); ^3^SAAHS unweighted data.

Analyses were also undertaken to assess any differences between the SAAHS and the 2008 National Aboriginal and Torres Strait Islander Social Survey (NATSISS) [1] and differences determined in the same way. There were no differences found for those speaking English (86.5% and 90.0% respectively), or Aboriginal (11.8% and 9.2), as their main language, or recognising an area as their traditional Country or Homelands (83.9% compared to 80.1%). Almost half of respondents identified as current smokers (48.3% and 48.0%) and 24% experienced financial stress in the last 12 months, compared to 31.4% in the NATSISS.

Differences were found between the two surveys for those reporting living on their traditional Country, ex- and non-smokers, housing tenure and self-assessed general health (Table 2). Additional measures from the SAAHS show that the prevalence of diabetes was 17.4%, 20.0% reported high blood pressure, and over three quarters of respondents reported having identified as Aboriginal at their last visit to a health service, if asked.

**Table 2:** South Australian Aboriginal Health Survey (SAAHS)^1^ compared to the National Aboriginal and Torres Strait Islander Social Survey (NATSISS)^2^ (where possible), SA Total, aged 15 years and over

	**SAAHS** ^**1**^ **(2010–2011)**			**NATSISS** ^**2**^ **(2008)**		
	**n**	**%**	**(95% CI)**	n*	**%**	**(95% CI)**
***Socio-demographic features***						
**Identification at last visit to an health service**						
Yes, identified as Aboriginal when asked	310	77.6	(73.4 – 81.5)	-	-	
No, chose to identify as Aboriginal	35	8.8	(6.4 – 12.0)	-	-	
Never been asked	49	12.2	(9.4 – 15.9)	-	-	
Not stated	5	1.4	(0.5 – 2.9)	-	-	
**Recognition of traditional lands**						
Yes	335	83.9	(81.1 – 88.2)	14,400	80.1	na
*Living on traditional land*	*105*	*31.3*	*(26.6 – 36.4)*	*3,200*	ǂ *17.9*	*(11.2 – 21.3)*
*Not living on traditional land*	*230*	*68.7*	*(63.5 – 73.4)*	*11,200*	ǂ *62.2*	(56.3 – 65.2)
No	47	11.8	(9.1 – 15.6)	3,600	ǂ 19.9	(14.3 – 22.7)
Not stated	17	4.2	(2.7 – 6.8)	-	-	
**Main language spoken at home**						
Aboriginal/Aboriginal English	51	11.8	(9.9 – 16.4)	1,600	ǂ 9.2	(5.4 – 11.1)
English (includes ‘Other’ in NATSISS)	345	86.5	(82.8 – 89.5)	16,300	90.8	(87.1 – 92.7)
*Speaks Aboriginal language*	*68*	*19.8*	*(16.0 – 24.4)*	*4,600*	*25.9*	(20.7 – 28.5)
*Speaks some words of language*	*167*	*48.3*	*(43.1 – 53.6)*	*7,200*	*40.1*	(34.6 – 42.9)
*No Aboriginal spoken*	*104*	*30.2*	*(25.6 – 35.3)*	*6,100*	*34.0*	(28.9 – 36.6)
*Not stated*	*6*	*1.7*	*(0.8 – 3.7)*	*-*	*-*	
**Housing tenure**						
Owner (with or without mortgage)	29	7.3	(5.1 – 10.2)	5,500	ǂ 30.4	na
Renter	170	42.4	(37.8 – 47.5)	12,200	67.9	(63.1 – 70.3)
Aboriginal housing scheme (rent or buy)	39	9.8	(7.2 – 13.1)	-	-	
Living at someone else’s house	98	24.5	(20.6 – 29.0)	-	-	
Other (includes Not Stated)	64	16.0	(12.8 – 20.0)	300	ǂ 1.7	(0.5 – 2.3)
**Financial stress in last 12 months**						
Ran out of money for basic living expenses	95	24.0	(19.9 – 28.2)	5,600	ǂ 31.4	(25.7 – 34.3)
Did not run out of money for basic living expenses	282	70.9	(66.0 – 74.9)	12,200	68.0	(62.4 – 70.9)
Not stated	22	5.2	(3.7 – 8.2)	-	-	
***Health status and risk factors***						
**Self-assessed health status**						
Excellent/Very Good	225	56.2	(51.5 – 61.2)	6,800	37.8	(33.5 – 40.0)
Good	103	25.8	(21.8 – 30.3)	6,300	35.2	(30.1 – 37.8)
Fair/Poor	71	18.0	(14.4 – 21.8)	4,900	ǂ 27.1	(22.7 – 29.3)
**Diabetes**						
Yes, doctor diagnosed/self-report	69	17.4	(14.0 – 21.4)	-	-	
No	327	82.1	(78.0 – 85.5)	-	-	
**High Blood Pressure**						
Yes, doctor diagnosed/self-report	80	20.0	(16.3 – 24.2)	-	-	
No	314	48.8	(74.5 – 82.5)	-	-	
Not stated	5	1.2	(0.5 – 2.9)	-	-	
**Smoking status**						
Current smoker	193	48.3	(43.4 – 53.2)	8,600	48.0	(43.1 – 50.5)
Ex-smoker	26	6.5	(4.5 – 9.4)	3,300	18.3	(15.1 – 19.9)
Non-smoker (never smoked)	173	43.3	(38.6 – 48.3)	6,100	33.7	(28.7 – 36.2)
**Total**	**399**	**100.0**		**17,900**	**100.0**	

Note: The weighting of data can result in rounding discrepancies or totals not adding *figures rounded to nearest 100.

ǂEstimate has a relative standard error of 25% to 50% and should be viewed with caution.

na – Relative Standard Error not available due to collapsing categories for comparison.

Sources: ^1^NATSISS (SA including APY Lands. Using Remoteness category classifications); ^2^SAAHS weighted data (does not include APY Lands).

## Discussion

This study has provided a representative sample of randomly chosen SA Aboriginal adults which can now be used for population analyses. The process presented provides a unique approach to data collection in Aboriginal populations, based upon methodologies used elsewhere [34,40] and adapted for local use.

### Cultural protocol

There are complex and culturally-specific reasons as to why Aboriginal recruitment and retainment for research purposes differs from mainstream and these are documented elsewhere [38,45]. We found retention of interviewers a major challenge and the inability to keep the interviewers engaged with the work, especially initially when they were employed to find eligible dwellings as well as interview, was very difficult. It was felt by some of the interviewers that the data collection methods were too restrictive and that some questions were repetitive and possibly inappropriate for Aboriginal people to answer fully. Certain language proved difficult to understand, and interviewers spoke of long, drawn out interviews with participants, and participants wanting to tell the ‘whole story’ to the interviewers, rather than giving a concise yes/no response. To address these challenges, and to benefit from feedback ourselves, the SAAHS research team initiated regular debriefing meetings, to issue paperwork and information, and to discuss any problems and concerns; however, it was difficult to get interviewers to attend these meetings, especially if they were not already actively interviewing and/or were located in rural or remote areas.

Recruitment of interviewers relied very much on Aboriginal networks. Email messages were sent around a large Aboriginal online network followed by a series of word-of-mouth referrals. Initially, interviewers were recruited solely on their interest and where they lived and each given their local area to door-knock. Interviewers provided feedback that in most cases respondents were comfortable being interviewed by someone from their own community, and even someone they knew. When interviewers knew the areas and/or communities they were visiting they were well placed to consult with the community. This knowledge of local community protocols, family structures and connections provided the interviewer with a sound basis for engagement with participants. Community collaboration is not a given; respectful communication, repeated contact, and establishment of trust is necessary for a successful outcome.

### Procedural challenges

It is well documented that Aboriginal populations are transient [46,47]. There were challenges with using census data that was more than four years old to identify where Aboriginal people lived and this was a severe limiting factor in identifying an initial eligible sample. Significant error was encountered in numbers of Aboriginal URs in each CD due in part to changing demography of the CD from the last census. Basing the population sampling strategy on more recent statistics, to obtain a representative sample in practice as well as in theory, would have improved our success rate of finding Aboriginal dwellings.

Although an arduous and financially challenging task, the decision to keep true to evidence based methodological principles provided a major success point for this project. Limitations arose from excluding CDs where there were less than ten Aboriginal URs reported at the 2006 Census and where the ratio of Aboriginal persons to total dwellings in a particular CD was less than 0.05. This resulted in 26.5% of the total SA Aboriginal population living in private dwellings being ineligible for selection: 39.6% in metropolitan Adelaide, 25.9% in rural SA and 9% in the remote parts of the state. Comparison between the eligible CDs and the ineligible CDs showed that the ineligible CDs had a higher proportion of persons living in metropolitan Adelaide and less living in rural and remote (see Table 1). These differences arise as a direct consequence of the methodology. The challenge of not having a fixed sampling frame initially meant that a high percentage of the project resources were used in finding eligible Aboriginal adults. By limiting the CDs we included as eligible, this enabled us to target the areas more densely populated with Aboriginal persons therefore increasing our chances of finding eligible participants. This however resulted in excluding those persons living in areas covered by the ineligible CDs and technically, we cannot claim that the sample represents them. What we think we can argue though, is that the sample achieved resembles the total SA population of Aboriginal adults (excluding the APY Lands), and therefore it is unlikely that these exclusions introduced bias into the sample. An obvious limitation to the SAAHS however, is the lack of data for the APY Lands and this limits the results, which cannot be extrapolated to the full SA Aboriginal adult population. However, it should be highlighted that consultation and the reaching of an agreement on how, and if, research is to be conducted, is an extremely important part of Aboriginal research and community priorities should always be respected. The fact that data collection did not go ahead in these areas, and cultural protocols were followed, is considered a strength of the methodology.

Another limitation of the data results from not achieving the estimated minimum sample size of 527; the final sample was 399. This was partly due to data collection not including any participants from the APY Lands where it had been estimated that the ten communities would have provided ten participants each; a possible total sample of 100. Additionally, contacting eligible persons identified from the door-knocking was hindered by a time lag between initial identification by non-Aboriginal researchers and contact by an Aboriginal interviewer and this led to further sample loss. With some people having moved or simply becoming unavailable, and finances constraining our ability to revisit some areas, some willing participants were not interviewed. Financial constraints also limited our ability to return to areas in remote and rural regions to complete the identification of eligible persons. A range of community factors can limit access to communities for research purposes; for example, Sorry Business will take precedence in communities. During the SAAHS data collection this led to further sample loss.

### Reliability limitations

Although the final sample size was sufficient to report on prevalence estimates at regional level, sub-analysis of specific target groups was not possible. In many cases, confidence intervals are wide due to small numbers and therefore prevalence estimates should be viewed with caution. To further explore the reliability of estimates, a comparison between the SAAHS and the 2008 NATSISS was undertaken showing some differences and some similarities (Table 2). Small numbers again result in many of the NATSISS comparative estimates needing to be viewed with caution. There were differences found between the two surveys for respondents reporting living on their traditional Country, housing tenure, self-assessed general health, and ex- and non-smoking (Table 2). For all of these estimates (except smoking) the ABS urge caution interpreting these data due to large relative standard error. Another reason for these differences may be explained by examining the methodological differences for the each of the surveys. In contrast to the NATSISS, the SAAHS was a small, single state survey undertaken by only Aboriginal interviewers who were often known to the participant. All questions in the SAAHS included “don’t know” and “refused” options. These categories in the NATSISS were not available in the data we had access to. It may be that some of the differences are hidden in these additional categories. However, it has been shown that the final dataset closely reflects the ratio of males to females, and the age structure of the SA Aboriginal adult population (excluding the APY Lands) as reported in the 2006 Census (Table 1) and data are weighted to account for the differences between regions.

In the lead-up to the release of the National Aboriginal and Torres Strait Islander Health Survey (NATSIHS) results [48], these data hold merit in that they explore wider determinants of health with respect to a number of cultural factors, such as access to, and discrimination from, health services, identification as Aboriginal when attending health services, health literacy, differentiating between Aboriginal Community Controlled Health Services (ACCHS) and SA Health managed services and reasons for, and barriers to, quitting smoking. A ‘Caring for Country’ Instrument was also included that has been shown to have significant health benefits for Aboriginal people in other states. The inclusion of these cultural determinants of health provides data that are not collected elsewhere for the SA Aboriginal adult population.

The attempt to de-cluster the sample by limiting the number of eligible persons per CD further limited the eligible sample where predicted numbers in a CD were lower than expected. On reflection, acknowledging that the most challenging areas to identify the Aboriginal population were the metropolitan areas, we should have aimed for a higher number of eligible persons in each metropolitan CD and possibly only four or five in rural and remote CDs. Over a quarter of the sample came from households where more than two persons were selected (n = 113) and 73% (n = 82) of these come from rural and remote areas. Data collection in these areas was mainly undertaken in Aboriginal communities and CDs were randomly distributed throughout the state. Therefore, we feel that the clustering resulting from the decision to interview all eligible persons in the household was compensated for by the distribution of the CDs, providing a distribution commensurate with the 2006 Census distribution of non-metropolitan CDs (39.6% for rural and 21.1% for remote).

## Conclusion

This paper describes the methodology, both culturally and statistically sound, used to obtain a representative population-based sample of randomly chosen Aboriginal adults for a cross-sectional health survey. The authors acknowledge that research methods for data collection in Aboriginal populations differ from those used for non-Aboriginal data collection and are subject to strict guidelines regarding cultural competence, safety, and respect [38]. It has been shown that by keeping to strict epidemiological principles while showing respectful engagement with community and following cultural protocols, random sampling in Aboriginal populations is achievable. We believe that the SAAHS provides a strong platform from which to continue this research.

## Abbreviations

ABS: Australian Bureau of Statistics
AC: Advisory committee
ACCHS: Aboriginal community controlled health service
APY: Anangu Pitjantjatjara Yankunytjatjara
CD: Collection district
NATSISS: National Aboriginal and Torres Strait Islander Social Survey
NATSIHS: National Aboriginal and Torres Strait Islander Health Survey
SA Health: South Australian Department for Health and Ageing
SA: South Australia
SAAHS: South Australian Aboriginal Health Survey
SPSS: Statistical package for the social sciences
UR: Usual resident
WAACHS: Western Australia Aboriginal Child Health Survey
